# QTL Mapping and Candidate Gene Analysis for Cotton Fiber Quality and Early Maturity Using F_2_ and F_3_ Generations

**DOI:** 10.3390/plants14071063

**Published:** 2025-03-29

**Authors:** Xiaoyun Jia, Jijie Zhu, Hongxia Zhao, Linglei Kong, Shijie Wang, Miao Li, Guoyin Wang

**Affiliations:** Institute of Cereal and Oil Crops, Hebei Academy of Agriculture and Forestry Sciences/Hebei Key Laboratory of Crop Genetics and Breeding, Shijiazhuang 050035, China; jiaxiaoyun1987@163.com (X.J.); nkyzhujj@163.com (J.Z.); jifengzhhx@163.com (H.Z.); linglei_kong@foxmail.com (L.K.); wgy1963@vip.sina.com (G.W.)

**Keywords:** upland cotton, fiber quality, early maturity, correlation analysis, quantitative trait loci, candidate gene

## Abstract

Cotton is the most important natural fiber-producing crop globally. High-quality fiber and early maturity are equally important breeding goals in the cotton industry. However, it remains challenging to synchronously improve these traits through conventional breeding techniques. To identify additional genetic information relating to fiber quality and early maturity, 11 phenotypic traits for the F_2_ and F_3_ generations were tested, and quantitative trait loci (QTL) mapping was performed. Candidate genes were analyzed using published RNA-seq datasets and qRT-PCR assays. All 11 tested traits showed bi-directional transgressive segregation, and most traits followed an approximately normal distribution. Overall, significant positive and significant negative correlations were observed among these traits. During cotton breeding, varieties with strong boll-setting ability can be selected from early-maturing materials that have high-quality fiber. A total of 102 QTLs were mapped, including 4 major and 3 stable QTLs. qFL-D13-1 was mapped in both the F_2_ and F_3_ generations, achieving a 3.94% to 11.39% contribution rate to the phenotypic variation. Three genes located in the QTL regions were identified based on their high expression levels in the three evaluated RNA-seq datasets. *Ghir_A04G014830.1*, covered by qHNFFB-A4-1 and qFU-A4-1, encoded ACLA-1. *Ghir_D13G015010.1*, encoding VTC2, and *Ghir_D13G016670.1*, encoding GA2OX1, were in the stable QTL qFL-D13-1 region. The qRT-PCR results suggested that these three genes may be involved in regulating seed development, fiber initiation, and fiber elongation. Overall, these findings contribute additional information for the breeding of high-yield, high fiber quality, and early-maturity varieties, as well as serve as a foundation for research on the underlying molecular mechanisms.

## 1. Introduction

Among the 45 diploid species (2n = 2× = 26) and 7 tetraploid species (2n = 4× = 52) of cotton, *Gossypium hirsutum* (upland cotton) is the most widely cultivated worldwide [1]. Upland cotton is the most important source of natural fiber for the textile industry and accounts for 95% of the world’s cotton production [2]. Furthermore, several by-products of cotton, such as fuzz fiber, cottonseed oil, and protein, are beneficial to human health, can serve as feed for animals, and have important uses in several industries [3,4,5]. China is the largest cotton producer (22–25%) and consumer (more than 7.5 million tons annually) in the world (http://www.fao.org/faostat/, accessed on 23 November 2024). Traditionally, achieving high yields in cotton production has been labor-intensive, especially in China, with increased mechanization being offered as one of the solutions for increasing cotton yield. Improving fiber quality and early maturity based on the existing high-yield varieties has become an important breeding goal, aiming to meet the requirements for high-quality textiles and mechanization. In practice, there is a complex antagonistic effect among high yield, high quality fiber, and early maturity, and how to coordinate the relationships among these three traits is thus an issue of paramount importance in current cotton breeding and production programs [6,7,8,9].

Most yield-, fiber quality-, and early-maturity-related traits are regulated by minor-effect genes and have complex correlations [6,7,8,9,10,11,12,13,14,15]. For yield traits, a significant positive correlation was found between boll weight (BW) and seed index (SI), a significant negative correlation was found between SI and lint percentage (LP), and a variable correlation was identified between BW and LP [6,16]. Regarding fiber quality traits, a significant positive correlation was detected between fiber length (FL) and fiber strength (FS), while micronaire (MC) was found to be significantly negatively correlated with FL and FS [6,17,18]. Significant positive correlations were found among early-maturity traits, such as plant height (PH), node of the first fruiting branch (NFFB), height of NFFB (HNFFB), flowering time (FT), flowering to boll-opening period (FBP), and whole growth period (WGP) [10,19,20]. For yield and fiber quality traits, significant positive correlations were found between SI with FL and between BW with MC, while significant negative correlations were found between LP with FS and between LP with FL [6,21]. For these three trait types, earlier maturity is achieved, and the lower the yield, the poorer the fiber quality [7]. Traditional breeding methods have been inefficient at simultaneously accomplishing high yield, high fiber quality, and early maturity.

To date, many QTLs relating to yield [22,23,24], fiber quality [17,25,26,27], and maturity [10,19,28] have been mapped. Studies simultaneously focusing on yield and fiber quality revealed the existence of negative genetic correlations between these two trait types [6,29]. Six important QTL clusters contained both yield- and fiber quality-related QTLs, and these exhibited opposing additive-effect directions [6]. For example, qClu-chr13-2 was associated with increased fiber quality but lower yield. Relatively few studies have concomitantly investigated cotton fiber quality and maturity [7,30]. Eleven chromosomal segments were found to concurrently harbor QTLs for yield, fiber quality, and early maturity; however, only three of these segments contained QTLs with the same additive-effect direction, while the QTLs in the other eight segments showed opposite additive-effect directions [7]. Thus, more work is needed to reveal the molecular mechanisms underlying these traits and leverage them to break the unfavorable linkage between high yield, high fiber quality, and early maturity.

Many candidate genes have been identified through QTL mapping and map-based cloning. For fiber quality, *Ghir_A10G022020* was proposed to act as a negative regulator of FL by interacting with NF-YA [31], whereas *GB_D11G3437*, *GB_D11G3460*, and *GB_D11G3471* were found to be associated with FS [32]. For early maturity, *GhAP1-D3* was reported to positively regulate flowering time and early maturity without affecting yield and fiber quality [33]. However, the current pool of genes known to be involved in the development of these traits in cotton cannot meet the needs of molecular design breeding.

In this study, to unearth additional information regarding the genetic relationships between high fiber quality and early maturity, a total of 11 traits were investigated in the F_2_ and F_3_ generations, and these traits were used for QTL mapping to identify QTLs based on a high-density genetic map [22]. Candidate genes for these traits were analyzed using published RNA-seq data and qRT-PCR assays. The results will provide a foundation for understanding the correlation between fiber quality and early maturity. And it also provides a method for breeding new cotton cultivars with high yield, high-quality fiber, and early maturity.

## 2. Results

### 2.1. Basic Statistics of the Phenotypic Traits

As shown in Table 1, JF173 had better fiber quality and early-maturity traits. All the tested traits showed bi-directional transgressive segregation in offspring populations. Additionally, most of the 11 traits followed an approximately normal distribution, as most of the absolute values of Kurt and Skew are less than 1 (Table 1).

### 2.2. Correlation Analysis

**Figure 1:**
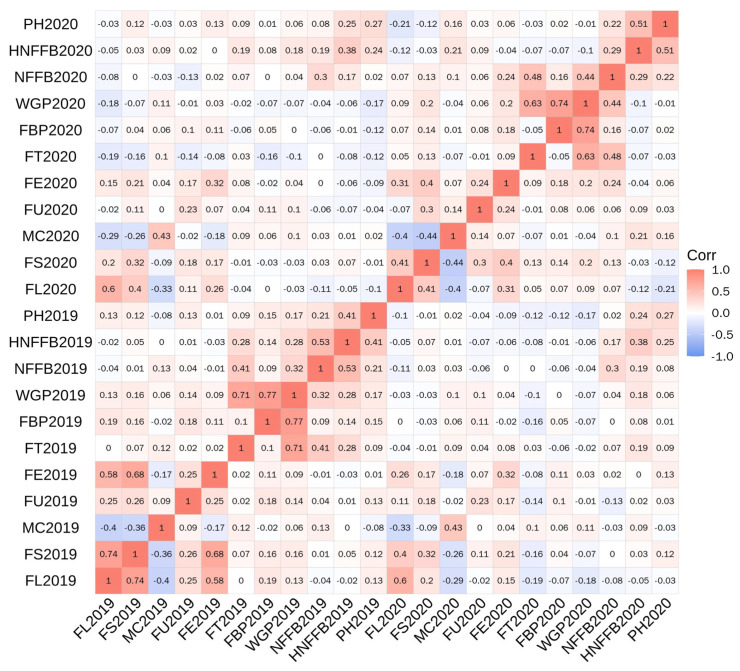
Heatmap of the correlation analysis among the tested traits in F_2_ and F_3_ generations. Note: FT, flowering time; FBP, flowering to boll-opening period; WGP, whole growth period; NFFB, node of the first fruiting branch; HNFFB, height of NFFB; PH, plant height; FL, fiber length; FS, fiber strength; MC, micronaire; FU, fiber uniformity; FE, fiber elongation rate.

Complex correlations were observed among the traits assessed (Table 2; Figure 1). Significant positive correlations were found between trait pairs, including some fiber quality trait pairs (FL–FS, FL–FE, FS–FE, and FU–FE) and all the maturity trait pairs. Additionally, the fiber quality–maturity trait pair FS–WGP also showed significant positive correlations. Significant negative correlations were found between the fiber quality trait pairs FL–MC and FS–MC. Thus, early-maturing materials can have high-quality fibers.

### 2.3. QTL Mapping Analysis

Based on the previously constructed high-density genetic map, 102 QTLs were mapped, including 11 for FL, 12 for FS, 8 for MC, 9 for FE, 7 for FU, 7 for FBP, 7 for FT, 6 for WGP, 10 for NFFB, 15 for HNFFB, and 10 for PH (Supplemental File S1). There were 42 QTLs that overlapped or were close to the positions of previously published QTLs. Four major-effect QTLs and three stable QTLs were found. Among these QTLs, qFL-D13-1 was mapped in the F2 and F3 populations, and its contribution rate to the PV ranged from 3.94% to 11.39%. This favorable allele was derived from JF173.

### 2.4. Candidate Gene Analysis

A total of 528 genes were obtained in the six above-mentioned chromosome regions (Supplemental File S2). KEGG pathway analysis indicated significant enrichment in 35 pathways involving 41 genes (Supplemental File S3). According to the published RNA-seq datasets of *G. hirsutum* cv. TM1 and *G. barbadense* cv. H7124 from the online software CottonMD, most of these 41 genes are expressed in vegetative or reproductive organs (Supplemental File S4). Three genes were identified based on their high expression levels in the evaluated RNA-seq datasets and their annotated functions.

**Figure 2:**
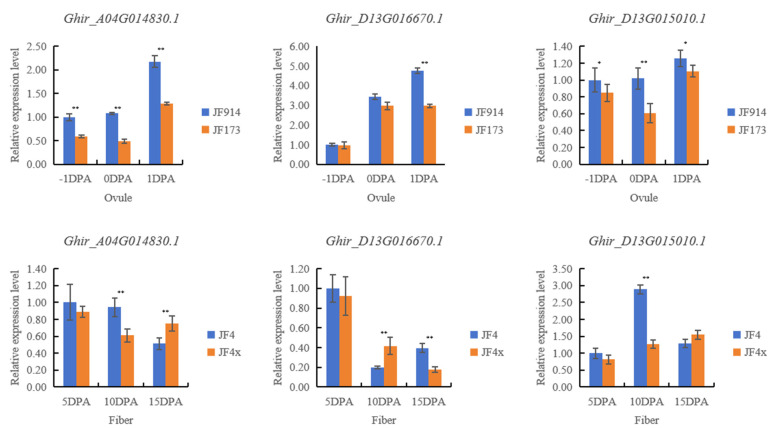
The expression levels of the three outstanding genes in ovules and fibers. Note: JF914, Jifeng 914; JF173, Jifeng 173; JF4, Jifeng 4; JF4x, Jifeng 4xuan. Values are means ± SD. *, *p* < 0.05; **, *p* < 0.01.

The qRT-PCR results revealed that the expression levels of these three genes showed continuous upregulation in ovules from −1 DPA to 1 DPA, and all exhibited higher expression levels in JF914 than in JF173 (Figure 2, Supplemental File S5). Meanwhile, these genes showed variable expression patterns among the fiber samples. For *Ghir_D13G015010.0*, the highest expression levels were observed in JF4 at 10 DPA. Compared with JF4x, the expression of *Ghir_A04G014830.1* in JF4 was relatively more stable and was expressed at higher levels at 5 DPA and 10 DPA; however, at 15 DPA, its expression was lower in JF4 than in JF4x. The expression of *Ghir_D13G016670.1* in fibers showed a steep decline from 5 DPA to 10 DPA, and its expression was higher in JF4 than in JF4x at 15 DPA.

## 3. Discussion

Cotton is the world’s most important natural fiber-producing crop. Breeding technology innovation plays a prominent role in the cultivation of new cotton varieties displaying high yield, high-quality fiber, and early-maturity traits that are also suitable for machine harvesting [9,34,35]. In China, in particular, the use of fully mechanized cultivation technology has necessitated the updating of cotton varieties. Owing to the long-standing dominance of high-yield breeding, coupled with the complex positive or negative correlations that exist among yield, fiber quality, and early-maturity traits, how to improve fiber quality and early maturity based on the existing high-yield varieties has become a major challenge for cotton production [8,35]. The main indicators for evaluating materials during breeding need to be adjusted. High yield is no longer the only assessment indicator or breeding goal. Fiber quality and early maturity become equally important traits as yield. In this study, both significant positive and significant negative correlations were observed among the fiber quality traits, while only significant positive correlations were observed among early maturity traits. Furthermore, a significant positive correlation was observed between FS and WGP. These results indicated that early-maturing plants with early flowering times and relatively long FBPs can produce high-quality fiber. To concomitantly achieve high yield, high-quality fiber, and early maturity in breeding, varieties with strong boll-setting ability can be selected from early-maturing materials that have high-quality fiber and medium single boll weight.

Thousands of QTLs relating to cotton yield, fiber quality, and early-maturity traits have been identified to date [6,36,37,38,39,40]. In this study, a total of 102 QTLs were mapped, including 42 that overlapped with published QTLs, which can prove the reliability of our results (Supplemental File S2). The other 60 QTLs might be newly identified in our populations, as the JF1271 and JF173 parents used in this study have not yet been used in fiber quality- and early-maturity-related QTL mapping studies. This also indicates that the parental materials we used have good genetic diversity. We did not find significance for certain QTLs such as early-maturity QTLs on D3 and fiber quality QTLs on D11. The main reason might be the use of parental materials and the marker density of the map. For example, there were only 39 SNPs on linkage group D3. We identified six chromosomal regions containing overlapping QTLs, which might provide additional genetic information allowing the unraveling of the complex correlations among the different traits. The stable QTL qFL-D13-1 overlapped with the QTL region reported previously using different populations [17], indicating that key genes may be located in this region.

Three candidate genes for the examined traits were identified in this study. *Ghir_A04G014830.1* is an ACLA-1-encoding gene covered by qFL-A4-1 and qBW-A4-1 [22]. ACLA-1 is a key enzyme in the production of acetyl-CoA from citrate during fatty acid synthesis and is also a positive regulator of secondary cell wall biosynthesis [41,42]. *Ghir_D13G015010.1*, encoding VTC2, and *Ghir_D13G016670.1*, encoding GA2OX1, were in the stable QTL qFL-D13-1 region. VTC has numerous functions in higher plants, such as regulating the cell cycle and modulating the synthesis of several hormones; moreover, cotton GhVTC1 was reported to be preferentially expressed in fiber from 5 DPA to 25 DPA, which is the dynamic elongation stage, and promotes cell elongation [43,44,45]. Gibberellin plays an important role in regulating cotton fiber initiation, while the overexpression of *GhGA2OX1* can increase the number of fiber initials [46,47]. The qRT-PCR results revealed that the three candidate genes showed continuously upregulated expression levels in ovules from −1 DPA to 1 DPA, and all three genes displayed higher expression levels in JF914 than in JF173. As JF914 has higher BW, SI, and LP than JF173, these genes may participate in the regulation of seed development and fiber initiation. In fiber samples, these genes may have functions in different fiber elongation stages. The highest expression of *Ghir_D13G015010.0* was detected in JF4 at 10 DPA, suggesting that it may promote fiber elongation at this stage. *Ghir_A04G014830.1* may exert positive effects on fiber elongation between 5 and 10 DPA. Meanwhile, *Ghir_D13G016670.1* may be involved in early fiber development. Further studies are needed to verify the functions of these genes.

## 4. Conclusions

In summary, a total of 102 QTLs relating to fiber quality and early-maturity traits were mapped in F_2_ and F_3_ generations. Based on the QTL mapping results and published RNA-seq datasets, three candidate genes were used for qRT-PCR assays. The results revealed that *Ghir_D13G015010.0*, *Ghir_A04G014830.1*, and *Ghir_D13G016670.1* may be involved in the regulation of seed development, fiber initiation, and elongation. And during cotton breeding, varieties with strong boll-setting ability can be selected from early-maturing materials that have high-quality fiber and medium single boll weight to concomitantly achieve high yield, high-quality fiber, and early maturity.

## 5. Materials and Methods

### 5.1. Plant Materials and Field Trials

**Figure 3:**
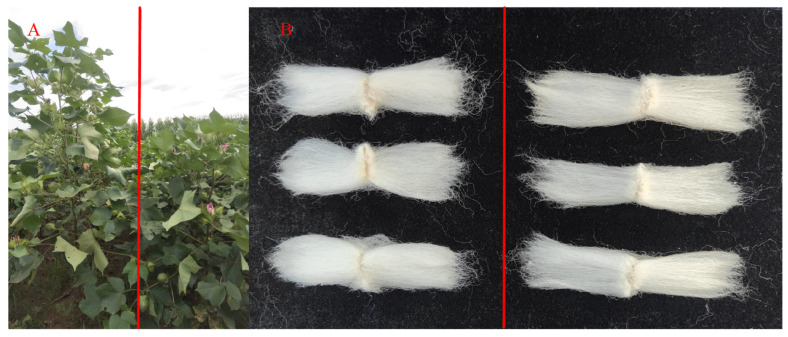
Phenotypes of the parents in the field. Note: (**A**), plant phenotype; (**B**), fiber. On the left side of the red lines is Jifeng 1271, and on the right side is Jifeng 173.

The high-yield cultivar, Jifeng 1271 (JF1271), served as the female parent, and the high-quality-fiber cultivar, Jifeng 173 (JF173), was used as the male parent (Figure 3). Compared to JF1271, JF173 has greater FL, greater FS, and better MC, and also matures approximately 10 days earlier. The F1 hybrid was obtained in the summer of 2018 at Shijiazhuang, Hebei province, and was self-pollinated the following winter at Sanya, Hainan province. The F_2_ generation, comprising 402 plants, was planted on 30 April 2019, at Shijiazhuang. The first 200 plants of the F_2_ generation that had not been damaged during the management process were selected for the construction of the high-density genetic map and self-pollinated to generate the F_3_ generation. The F_3_ generation, comprising 200 lines, was planted on 27 April 2020, at Shijiazhuang, and two rows were planted for each line. All tested materials were planted in 7 m long rows, with 0.7 m between adjacent rows and 0.2 m between adjacent plants. Field management was performed under local practices. The experimental site is loam soil, with cotton as the previous crop. The soil contained 35.1 mg/Kg organic matter content, 251.6 mg/Kg available potassium content, and 1.5 g/Kg total nitrogen content. Before sowing, 750 Kg/ha of cotton-specific fertilizer was used as the base fertilizer. The weather in 2019 was mainly characterized by high temperatures and drought, and in total, irrigation was applied 3 times (28 May, 2 July, and 4 September) and urea was applied 2 times (112.5 Kg/ha each on 1 and 29 July) throughout the entire growth period. The weather in 2020 was more suitable for cotton growth with no irrigation and with urea being applied 3 times (75 Kg/ha on 1 July, 120 Kg/ha on 26 July, and 90 Kg/ha on 19 August). To control pests and ensure the cotton’s normal growth, chemical control was conducted a total of 12 and 16 times in 2019 and 2020, respectively.

### 5.2. Trait Collection and Data Analysis

A total of 11 traits were investigated in this study, with 5 relating to fiber quality (fiber length [FL], fiber strength [FS], micronaire [MC], fiber uniformity [FU], and fiber elongation rate [FE]) and 6 relating to early maturity (flowering time [FT], flowering to boll-opening period [FBP], whole growth period [WGP], plant height [PH], node of the first fruiting branch [NFFB], and height of NFFB [HNFFB]). Phenotypic data were collected according to the following methods:

All bolls on the F_2_ plants were harvested and counted, and thirty naturally opened bolls from each F_3_ row, covering the upper, middle, and lower parts of the cotton plants, were hand-harvested. After ginning, the five fiber quality traits—FL, FS, FU, MC, and FE—were evaluated using a high-volume instrument (HFT9000) at the Cotton Quality Testing Center in the Institute of Cotton Research, Chinese Academy of Agricultural Science (CRI CAAS). Early-maturity traits were investigated using the following methods: FT was the number of days from the date of sowing to the date when 50% of the plants were flowering (for the F_2_ population, FT indicates the number of days from the date of sowing to the date when the first flower appeared); WGP was the number of days from the date of sowing to the date when 50% of the plants reached boll opening (for the F_2_ population, WGP indicates the number of days from the date of sowing to the date when the first boll opened); FBP was the number of days from flowering to boll opening; PH was the height from the ground to the main stem tip; NFFB was node number of the first fruiting branch, with the cotyledonary node recorded as zero; and HNFFB was the height from the ground to the NFFB. For the F_3_ generation, all the plants were used for the assessment of FT and WGP, and 10 plants in the middle of each row were used for the determination of PH, NFFB, and HNFFB. Mean values for the two rows represented the phenotype for each F_3_ line and were used for principal component analysis.

Basic statistics were calculated using Excel 2010, such as Max, Min, Mean, Kurt, Skew, and STDEV, and CV(%) = STDEV/Mean × 100.

### 5.3. QTL Mapping

A high-density genetic map was previously constructed for the F_2_ generation (Supplemental File S6). Briefly, a total of 383.07 Gb of sequencing data were obtained using genotyping by sequencing (GBS), with an average of 27.12 Gb and 9.64× depth in the parents and 1.64 Gb and 0.7× depth in the F_2_ plants. The Q30 score reached 95.77%. The genetic map contained 16,088 single-nucleotide polymorphisms (SNPs) and spanned a total of 4282.81 cM, ranging from 150.12 cM on D13 to 179.92 cM on D1. The SNPs were unevenly distributed in the 26 linkage groups, with D3 containing only 39 SNPs while D5 contained 1746 SNPs. Based on this genetic map, QTL mapping for the 16 tested traits was performed using QTL IciMapping 4.0 software [48]. The parameters were 1 cM per step and PIN = 0.001; a logarithm of the odds (LOD) score of 2.5 was used to determine a QTL. QTLs with a greater than 10% contribution rate to the phenotypic variation (PV) were classified as major-effect QTLs, while those that could be mapped in both the F_2_ and F_3_ generations were categorized as stable QTLs.

### 5.4. Candidate Gene Analysis

Gene information relating to the QTL regions was obtained with the online software COTTONFGD (https://cottonfgd.net/, accessed on 16 May 2024) using the *G. hirsutum* cv. TM1 HAU_v.1.1 sequence as a reference [49]. The expression levels of the annotated genes were analyzed using the online software CottonMD (https://yanglab.hzau.edu.cn/CottonMD/, accessed on 16 May 2024).

Gene expression patterns were analyzed by qRT-PCR using ovule samples (−1 day post anthesis [DPA], 0 DPA, and 1 DPA) from Jifeng 914 (JF914) and JF173 and fiber samples (5 DPA, 10 DPA, and 15 DPA) from Jifeng 4 (JF4) and Jifeng 4xuan (JF4x) as materials. These materials have the same ancestor, 97G1, as the parental materials in this study (Table 3). The differences in yield traits such as BW, SI, and LP between JF914 and JF173 are more significant than those between JF1271 and JF173. Additionally, JF4 and JF4x are sister lines with marked differences in fiber quality, especially FL, FS, and MC. In this study, given that the most important QTLs were those related to yield and fiber quality, samples from JF914, JF173, JF4, and JF4x were used for qRT-PCR assays.

To collect ovules and fibers, flowers blooming on the same day were marked. Ten plants with consistent growth for each material were selected, and one bud or one boll from the same part of each plant was picked. Pooled samples from 10 plants of each material were used for qRT-PCR. All the samples were immediately frozen in liquid nitrogen and stored at −80 °C before use. Total RNA was extracted using a Plant RNA Purification Kit (Tiangen, Beijing, China). First-strand cDNA was reverse-transcribed from 1 μg of total RNA using a FastKing gDNA Dispelling RT SuperMix Kit (Tiangen, Beijing, China). qRT-PCR was carried out with SYBR Premix Ex Taq (TAKARA, Osaka, Japan) on a LightCycler 480 Instrument (Roche, Rotkreuz, Switzerland), with three technical replicates per sample. *GhHis3* was used as the internal reference gene. The relative expression of genes was analyzed by the 2^−ΔΔCt^ method. Mean values of the 3 replicates represented gene expression levels.

## Figures and Tables

**Table 1:** Basic statistics of the observed traits in the parents and offspring.

Trait	Population	Parents		Offspring					
		Jifeng1271	Jifeng173	Max	Min	Mean	CV/%	Kurt	Skew
FT/d	F_2_	66.00	63.00	72.00	61.00	65.23	3.60	−0.42	0.54
	F_3_	73.00	70.00	78.00	66.00	72.42	2.59	0.94	0.27
FBP/d	F_2_	51.00	45.00	53.00	41.00	46.65	5.71	−0.38	0.15
	F_3_	54.00	49.50	59.00	46.00	51.60	4.18	0.62	0.55
WGP/d	F_2_	117.00	108.00	122.00	105.00	111.88	3.32	−0.50	0.10
	F_3_	127.00	119.50	132.00	117.00	124.02	2.24	−0.04	0.53
NFFB/cm	F_2_	7.00	5.30	8.00	5.00	6.50	13.10	−0.59	0.19
	F_3_	7.20	5.70	8.10	5.20	6.74	7.47	0.09	0.12
HNFFB/cm	F_2_	29.90	19.50	33.00	14.00	23.24	15.52	−0.08	−0.21
	F_3_	21.65	16.03	23.56	15.80	19.45	7.55	−0.20	0.15
PH/cm	F_2_	103.00	74.40	97.00	51.00	79.66	9.22	1.23	−0.58
	F_3_	108.25	78.20	114.40	81.50	93.80	6.01	0.29	0.57
FL/mm	F_2_	27.00	29.90	32.10	24.60	28.65	3.82	1.06	0.14
	F_3_	29.80	32.85	32.60	27.70	30.83	3.00	0.67	−0.68
FS/cN·tex-1	F_2_	25.00	31.40	34.00	23.80	29.49	5.38	0.47	0.02
	F_3_	26.20	32.40	35.20	27.00	30.66	4.47	−0.12	0.20
MC	F_2_	5.50	4.00	5.90	3.90	5.17	5.61	1.17	−0.67
	F_3_	5.45	4.10	5.80	4.00	4.94	7.40	−0.47	−0.22
FU/%	F_2_	83.80	86.20	87.20	81.50	84.76	1.20	0.67	−0.52
	F_3_	83.20	85.25	87.00	81.70	84.69	1.12	−0.14	−0.21
FE/%	F_2_	6.80	6.70	6.90	6.60	6.76	0.85	−0.34	−0.21
	F_3_	6.75	6.75	7.00	6.60	6.77	1.17	−0.02	0.11

Note: FT, flowering time; FBP, flowering to boll-opening period; WGP, whole growth period; NFFB, node of the first fruiting branch; HNFFB, height of NFFB; PH, plant height; FL, fiber length; FS, fiber strength; MC, micronaire; FU, fiber uniformity; FE, fiber elongation rate.

**Table 2:** Correlation analysis among the tested traits.

Trait	Population	FS	MC	FU	FE	FT	FBP	WGP	NFFB	HNFFB	PH
FL	F_2_	0.74 **	−0.41 **	0.25 **	0.58 **	0	0.19 **	0.13	−0.03	−0.02	0.13
	F_3_	0.41 **	−0.41 **	−0.07	0.30 **	0.06	0.07	0.10	0.08	−0.13	−0.20 **
FS	F_2_	1	−0.38 **	0.26 **	0.68 **	0.07	0.16 *	0.16 *	0.01	0.05	0.12
	F_3_	1	−0.45 **	0.29 **	0.39 **	0.15 *	0.13	0.20 **	0.14 *	−0.05	−0.12
MC	F_2_		1	0.09	−0.17 *	0.12	−0.02	0.06	0.12	0	−0.09
	F_3_		1	0.11	0.08	−0.08	0.01	−0.04	0.09	0.22 **	0.13
FU	F_2_			1	0.25 **	0.02	0.18 *	0.14 *	0.04	0.01	0.14
	F_3_			1	0.22 **	0	0.06	0.04	0.06	0.07	0.04
FE	F_2_				1	0.02	0.11	0.09	−0.02	−0.03	0
	F_3_				1	0.08	0.19 **	0.20 **	0.24 **	−0.04	0.03
FT	F_2_					1	0.1	0.70 **	0.41 **	0.27 **	0.08
	F_3_					1	−0.05	0.63 **	0.49 **	0.08	−0.05
FBP	F_2_						1	0.78 **	0.09	0.08	0.12
	F_3_						1	0.74 **	0.15 *	−0.07	0.02
WGP	F_2_							1	0.32 **	0.23 **	0.13
	F_3_							1	0.45 **	0.11	−0.02
NFFB	F_2_								1	0.52 **	0.20 **
	F_3_								1	0.28 **	0.19 **
HNFFB	F_2_									1	0.41 **
	F_3_									1	0.49 **

Note: FT, flowering time; FBP, flowering to boll-opening period; WGP, whole growth period; NFFB, node of the first fruiting branch; HNFFB, height of NFFB; PH, plant height; FL, fiber length; FS, fiber strength; MC, micronaire; FU, fiber uniformity; FE, fiber elongation rate; *, *p* < 0.05; **, *p* < 0.01.

**Table 3:** The parental combinations of the materials used for qRT-PCR.

Material	Parental Combination	BW/g	LP/%	SI/g	FL/mm	FS/cN·tex-1	MC
Jifeng 1271	Jimian 20 × 97G1	6.3	37.3	10.9	29.0	30.4	5.0
Jifeng 173	(99-68 × 97G1) × Jimian 20	5.3	35.5	11.7	32.7	35.5	4.5
Jifeng 914	Ji 668 × 97G1	7.4	40.1	12.2	31.0	31.9	4.9
Jifeng 4	97-668 × 97G1	6.9	40.8	12.9	32.0	29.1	5.6
Jifeng 4xuan	97-668 × 97G1	7.3	40.3	12.4	28.7	29.6	5.3

Note: BW, boll weight; LP, lint percentage; SI, seed index; FL, fiber length; FS, fiber strength; MC, micronaire.

## Data Availability

Data are contained within the article or Supplementary Materials.

## Supplementary Materials

The following supporting information can be downloaded at: https://www.mdpi.com/article/10.3390/plants14071063/s1, Supplemental File S1. QTL. Supplemental File S2. annotated gene. Supplemental File S3. KEGG. Supplemental File S4. gene expression in RNA-seq. Supplemental File S5. qRT-PCR. Supplemental File S6. genetic map. Refs. [50,51] are cited in the Supplementary Materials.

## Abbreviations

QTL: quantitative trait loci; PV, phenotypic variation; FL, fiber length; FS, fiber strength; MC, micronaire; FU, fiber uniformity; FE, fiber elongation rate; FT, flowering timing; WGP, whole growth period; FBP, flowering to boll-opening period; NFFB, node of first fruiting branch; HNFFB, height of NFFB; PH, plant height.
